# Cancer risk in information technology workers: a UK Biobank study

**DOI:** 10.1093/occmed/kqad070

**Published:** 2023-07-21

**Authors:** D Lalloo, J Lewsey, S V Katikireddi, E B Macdonald, E Demou

**Affiliations:** Healthy Working Lives Group, Clarice Pears Building, School of Health & Wellbeing, University of Glasgow, 90 Byres Road, Glasgow, G12 8TB, UK; MRC/CSO Social and Public Health Sciences Unit, Clarice Pears Building, School of Health & Wellbeing, University of Glasgow, 90 Byres Road, Glasgow, G12 8TB, UK; Health Economics and Health Technology Assessment, Clarice Pears Building, School of Health & Wellbeing, University of Glasgow, 90 Byres Road, Glasgow, G12 8TB, UK; MRC/CSO Social and Public Health Sciences Unit, Clarice Pears Building, School of Health & Wellbeing, University of Glasgow, 90 Byres Road, Glasgow, G12 8TB, UK; Healthy Working Lives Group, Clarice Pears Building, School of Health & Wellbeing, University of Glasgow, 90 Byres Road, Glasgow, G12 8TB, UK; MRC/CSO Social and Public Health Sciences Unit, Clarice Pears Building, School of Health & Wellbeing, University of Glasgow, 90 Byres Road, Glasgow, G12 8TB, UK

## Abstract

**Background:**

The information technology (IT) workforce has been growing more rapidly than others, with occupational health (OH) risks of sedentary behaviour, physical inactivity and poor diet, yet studies of their non-communicable disease risk, notably cancer, are lacking.

**Aims:**

To investigate cancer risk in IT workers compared to others in employment and the nine major Standard Occupational Classification (SOC) groups.

**Methods:**

We evaluated incident diagnosed cancers in the UK Biobank cohort through national cancer registry linkage. Cox proportional hazard regression models, with 15-year follow-up, were used to compare incident cancer risk among IT workers with all other employed participants and with the nine major SOC groups.

**Results:**

Overall, 10 517 (4%) employed participants were IT workers. Adjusting for confounders, IT workers had a slightly lower cancer incidence compared to all other employed participants (Model 2: hazard ratio = 0.91, 95% confidence interval [CI] 0.83–1.01). Compared to the nine major SOC groups, they had a similar (Major Groups 2, 5 and 8) or lower (Major Groups 1, 3, 4, 6, 7 and 9) cancer incidence.

**Conclusions:**

Despite their occupational risks of sedentary behaviour, poor diet and physical inactivity, IT workers do not have an increased cancer incidence compared to all other employed participants and the nine major SOC groups. This study paves the way for large, longitudinal health outcome studies of this under-researched and rapidly growing occupational group.

## Teaser

The information technology (IT) workforce is growing more rapidly than others, accounting for almost 10% of the UK workforce, and despite their known occupational risks of sedentary behaviour, poor diet and physical inactivity, cancer outcomes among IT workers are unknown. In this study, we identified that IT workers do not have an increased cancer incidence compared to all other employed participants and the nine major Standard Occupational Classification groups.

**Key learning points**

**What is already known about this subject**

- The information technology revolution has seen a rapid growth in the information technology workforce, yet studies of their non-communicable disease risk, notably cancer, are lacking.
- To date, there are no published studies specifically investigating cancer risk in information technology workers.

**What this study adds**

- In this, the first study investigating cancer risk in information technology workers, despite their known occupational risks of sedentary behaviour, poor diet and physical inactivity, information technology workers did not have an increased cancer incidence compared to all other employed participants and the nine major Standard Occupational Classification groups.

**What impact this may have on practice, policy or procedure**

- While further research is needed to replicate our findings, this study sets a baseline in our understanding of information technology worker cancer risk and paves the way for further large, longitudinal health outcome studies in this occupational group.

## Introduction

Information technology (IT) workers are a skilled occupational group who perform any function related to IT or computer systems. Their roles include hardware, software, systems and network design/development/management, data management/processing, helpdesk assistance and, more recently, information security, ‘big data’ collection and artificial intelligence [1].

IT workers have a substantially higher occupational exposure risk for sedentary work compared to the general working population (five times higher) and similar comparable occupations [2]. Poor diet [3] and reduced physical activity [4] have also been reported in small, localized studies.

Prolonged sedentary behaviour is positively associated with several cancers, including colorectal, breast and endometrial [5], and higher occupational sedentary behaviour with colon and rectal cancers [6]. Physical activity is strongly associated with lower cancer risk [5] and poor diet with increased cancer risk [7].

Cancer is a leading cause of morbidity and mortality, responsible for nearly one in six deaths worldwide [8]. Around 30–50% of cancers are believed to be preventable through healthier lifestyles [8]. High sedentary behaviour [2], poor diet [3] and reduced physical activity [4] in IT workers may impact their cancer risk yet to date, no studies have explored this. This study aims to address that knowledge gap. We evaluated incident cancer in IT workers compared with (i) the general working population and (ii) the nine major standard occupational classification (SOC) groups over a 15-year period and examined whether socio-demographic, lifestyle and occupational factors modify that association. This research is particularly relevant given the IT workforce is growing more rapidly than others [9], accounting for almost 10% of the UK workforce [9].

## Methods

We conducted a population-based cohort study using UK Biobank with national cancer registry data linkage. UK Biobank is a large cohort study from across Great Britain of over 502 000 participants (6% response rate) aged 37–73 years recruited between 2006 and 2010. This entailed touch-screen questionnaire completion and face-to-face interviews with physical and biological measurements, described in detail elsewhere (https://www.ukbiobank.ac.uk/) [2]. Baseline assessment included socio-demographic, health behaviour and lifestyle data, physical measurements and employment status [2] (using SOC V.2000).

Our study population comprised IT workers and all other employed Biobank participants. Within the latter group, we categorized the nine major SOC groups (Table 2) with IT workers excluded from their respective groups. Cancer outcomes were coded according to the International Classification of Diseases ICD-9 and ICD-10. The primary outcome was defined as a first episode/incident cancer diagnosis (see Table 1, available as Supplementary data at *Occupational Medicine* Online, for selection codes).

**Table 2.** Cox proportional hazard models of the association between socio-demographic factors and incident cancer^a^ in (a) IT workers compared to all other employed participants in the UK Biobank and (b) IT workers compared to the nine major category standard occupational classification (SOC) occupational groups

		Model 0^c^	Model 1^d^
		Unadjusted HR (95% CI)	Adjusted HR (95% CI)
a	Failures 13 351		
	All other employed participants Incidence rate^b^ (4.7)	1.00	1.00
	All IT workers	0.77	0.91
	Incidence rate^b^ (3.5)	(0.70–0.85)	(0.83–1.01)
b1	Failures 1082		
	All other managers, directors and senior officials Incidence rate^b^ (4.8)	1.00	1.00
	All IT workers	0.77	0.89
	Incidence rate^b^ (3.5)	(0.69–0.86)	(0.80–1.00)
b2	Failures 1405		
	All other professional occupations Incidence rate^b^ (4.7)	1.00	1.00
	All IT workers	0.76	0.97
	Incidence rate^b^ (3.5)	(0.68–0.84)	(0.87–1.08)
b3	Failures 1045		
	All other associate professional and technical occupations Incidence rate^b^ (4.4)	1.00	1.00
	All IT workers	0.81	0.90
	Incidence rate^b^ (3.5)	(0.73–0.90)	(0.80–1.00)
b4	Failures 715		
	Administrative and secretarial occupations Incidence rate^b^ (4.7)	1.00	1.00
	All IT workers	0.77	0.90
	Incidence rate^b^ (3.5)	(0.69–0.86)	(0.80–1.03)
b5	Failures 648		
	Skilled trades occupations Incidence rate^b^ (4.6)	1.00	1.00
	All IT workers	0.75	1.02
	Incidence rate^b^ (3.5)	(0.67–0.84)	(0.89–1.16)
b6	Failures 356		
	All IT workers caring, leisure and other service occupations	1.00	1.00
	Incidence rate^b^ (4.3)	0.84	0.94
	Incidence rate^b^ (3.5)	(0.74–0.95)	(0.80–1.10)
b7	Failures 293		
	Sales and customer service occupations Incidence rate^b^ (4.6)	1.00	1.00
	All IT workers	0.72	0.86
	Incidence rate^b^ (3.5)	(0.57–0.91)	(0.64–1.16)
b8	Failures 371		
	Process, plant and machine operatives Incidence rate^b^ (5.2)	1.00	1.00
	All IT workers	0.71	0.98
	Incidence rate^b^ (3.5)	(0.63–0.80)	(0.84–1.14)
b9	Failures 385		
	Elementary occupations Incidence rate^b^ (4.9)	1.00	1.00
	All IT workers	0.70	0.92
	Incidence rate^*^ (3.5)	(0.62–0.79)	(0.78–1.07)

Longitudinal study population: all employed Biobank participants with linked national cancer registry data/records. Model 0^c^ = Unadjusted. Model 1^d^ = Model 0 + potential confounders/socio-demographic factors, that is, age, sex, ethnicity, deprivation index, educational attainment, assessment centre and date of assessment.

^a^Cancer registry data were available from 1957 onwards until 31 January 2021 for Scotland and from 1971 onwards until 29 February 2020 for England and Wales.

^b^Rates are expressed per 1000 and based on person-years.

Individuals who died were censored and not recorded as having an event. For each participant, follow-up commenced at the baseline UK Biobank assessment date (2006–2010) and ended on the cancer registry end dates (Table 2) unless preceded by date of death, or date of a first cancer diagnosis.

Participants with a pre-existing cancer diagnosis at baseline or the preceding years of the cancer registry, were excluded from the analysis (*n* = 14 352).

Having ascertained that the proportional hazards assumption had been met (using Kaplan–Meier plots), survival analyses for first/incident cancer outcomes were conducted using Cox proportional hazard regression.

Models were applied in a staged process; Model 0 was unadjusted for all covariates; Model 1 adjusted for potential confounders/socio-demographic factors; Model 2 additionally adjusted for potential mediators/lifestyle and occupational factors (see Table 2;Supplementary data at *Occupational Medicine* Online).

Analyses were performed using Stata V17 (StataCorp LP). Multiple imputation by chained equations was used to impute missing data, creating 20 imputation datasets. This study was conducted under generic UK Biobank approval from NHS National Research Ethics service (Ref 11/NW/0382), Application number 17333.

## Results

The analytical cohort comprised 272 733 employed participants, of which 10 517 (4%) were IT workers (Table 1; Figure 1, available as Supplementary data at *Occupational Medicine* Online). Over three-quarters of IT workers (77%) were male, with a median age of 50 years (25th/75th percentile: 45/55). Demographics of the nine major SOC groups are presented in Table 2 (available as Supplementary data at *Occupational Medicine* Online).

**Table 1.** Socio-demographic, lifestyle and work characteristics in IT workers compared to all other employed participants in the UK Biobank with cancer registry data linkage

Total *n* (%)	All other employed, *n* (%)	All IT workers, *n* (%)
272 733 (100)	262 216 (96)	10 517 (4)
Socio-demographic		
Sex		
Male	124 101 (47)	8074 (77)
Female	138 115 (53)	2443 (23)
Age (years), median (IQR; Q1/Q3)	53 (11; 47/58)	50 (10; 45/55)
Age (years)		
40–44^a^	41 192 (16)	2593 (25)
45–49	52 864 (20)	2622 (25)
50–54	58 279 (22)	2464 (23)
55–59	58 116 (22)	1782 (17)
60–64	41 107 (16)	917 (9)
65+	10 658 (4)	139 (1)
Ethnicity		
White	245 433 (94)	9876 (94)
Non-White	15 982 (6)	609 (6)
Missing^b^	801 (0)	32 (0)
Townsend deprivation index		
1 (least deprived quintile)	116 928 (45)	5478 (52)
2	59 708 (23)	2331 (22)
3	40 460 (15)	1470 (14)
4	31 734 (12)	931 (9)
5 (most deprived quintile)	13 010 (5)	296 (3)
Missing^b^	376 (0)	11 (0)
Household annual income (£)		
Less than £18 000	25 538 (10)	197 (2)
£18 000–£30 999	53 029 (20)	846 (8)
£31 000–£51 999	73 931 (28)	3130 (30)
£52 000–£100 000	65 348 (25)	4552 (43)
Greater than £100 000	17 205 (7)	1091 (10)
Missing^b^	27 165 (10)	701 (7)
Highest qualification		
Degree	96 185 (37)	6093 (58)
HNC/HND	18 049 (7)	560 (5)
School	106 094 (40)	3518 (34)
Other	11 629 (4)	96 (1)
None of the above	26 422 (10)	132 (1)
Missing^b^	3837 (1)	118 (1)
Lifestyle		
Body mass index (kg/m^2^)		
<25	89 860 (34)	3531 (34)
≥25	171 314 (65)	6958 (66)
Missing^b^	1042 (0)	28 (0)
Smoking status		
Never smoker	149 879 (57)	6589 (63)
Previous/Current smoker	111 598 (43)	3911 (37)
Missing^b^	739 (0)	17 (0)
Alcohol consumption^d^ (units/week)		
≤14	53 301 (20)	2100 (20)
>14	131 699 (50)	6027 (57)
Missing^b^	77 216 (30)	2390 (23)
Physical activity (MET min/week)		
<600	24 888 (9)	1370 (13)
≥600	112 457 (43)	4512 (43)
Missing^b^	124 871 (48)	4635 (44)
Total raw or cooked fruit/vegetables (portions/day)		
<5	54 519 (21)	2552 (24)
≥5	201 520 (77)	7867 (75)
Missing^b^	6177 (2)	98 (1)
Total screen-time outside work^c^ (h/day)		
≤2	131 120 (50)	5830 (55)
>2	126 352 (48)	4564 (43)
Missing^b^	4744 (2)	123 (1)
Occupational		
Job involves shift work		
Never/rarely	214 803 (82)	9773 (93)
Always/usually/sometimes	46 730 (18)	738 (7)
Missing^b^	683 (0)	6 (0)
Job involves walking/standing		
Always/usually/sometimes	173 829 (66)	2705 (26)
Never/rarely	88 014 (34)	7809 (74)
Missing^b^	373 (0)	3 (0)

IQR, interquartile range; HNC, higher national certificate; HND, higher national diploma; MET, metabolic equivalent.

^a^35–39 year olds added to this total due to very small numbers, *n* = 2.

^b^Includes ‘missing’, ‘do not know’ and ‘prefer not to answer’ responses.

^c^Total screen-time estimated as the sum of computer screen-time outside work and TV viewing (h/day).

^d^Recommended alcohol consumption guidelines changed in 2016 (i.e. following baseline data collection) from 21 units/week for women and 28 units/week for men to current thresholds of 14 units/week for men and women.

The sample size for the survival analysis for incident cancer in IT workers compared to all other employed participants was 13 351 participants, with a median survival time of 11 years (Table 2).

After adjustment for confounders, compared to all other employed participants, IT workers overall have a slightly lower cancer incidence (Model 2: hazard ratio [HR] = 0.91, 95% confidence interval [CI] 0.83–1.01).

After adjustment for confounders, compared to all major SOC groups, IT workers had a similar (Major Groups 2, 5 and 8) or lower (Major Groups 1, 3, 4, 6, 7 and 9) cancer incidence (Table 2). In both cases, CIs were wide.

## Discussion

In this study, despite their known occupational risks of sedentary behaviour, poor diet and reduced physical activity, IT workers did not have an increased cancer incidence compared to all other employed participants and either of the major SOC groups. There are no published studies specifically evaluating cancer risk in IT workers to compare our results with, and further research using other cohorts is needed to replicate our findings. Longer follow-up studies are also needed, given the prolonged latency period of cancers.

This UK-based study is the first to examine cancer risk in IT workers, with a rich characterization of variables. It is not restricted to a single IT company or sector, providing a more generalizable overview of the risks of IT work.

Low response rates, healthy-worker effect and selection bias are potential limitations in UK Biobank, although studies suggest that risk factor associations in this cohort seem to be generalizable [10]. Lower numbers in some of the major SOC group comparisons reduced power and there were insufficient numbers in our dataset to investigate site-specific cancers. While we accounted for socio-economic factors/potential confounders in our models, residual confounding remains possible.

This study sets a baseline in our understanding of IT worker cancer risk. The methodology used (i.e. studying specific occupational groups using administrative and linked data) can be replicated for studies on other groups with similar or different occupational risk factors for cancer. This study also paves the way for further large, longitudinal studies to investigate other health outcomes in IT workers. This will have important implications for targeting and informing workplace interventions to mitigate risk.

## Supplementary Material

kqad070_suppl_Supplementary_Material

## Data Availability

Data may be obtained from a third party and are not publicly available.

## References

[1] Code Academy. What Is Information Technology (IT)? 2022. https://www.codecademy.com/resources/blog/what-is-information-technology/ (1 December 2022, date last accessed).

[2] Lalloo D , Lewsey J, Katikireddi SV, Macdonald EB, Demou E. Health, lifestyle and occupational risks in Information Technology workers. Occup Med (Lond). 2021;71:68–74. 33515462 10.1093/occmed/kqaa222 PMC8034523

[3] Nasui BA , Toth A, Popescu CA et al. Comparative study on nutrition and lifestyle of information technology workers from Romania before and during COVID-19 pandemic. Nutrients. 2022;14:1202. 35334858 10.3390/nu14061202 PMC8953937

[4] Dorner TE , Lackinger C, Haider S, Grabovac I, Stein KV. the influence of occupational categories on overall and domain-specific physical activity and the association with chronic diseases. An analysis using the Austrian Health Interview Survey. Int J Environ Res Public Health. 2021;18:2148. 33671784 10.3390/ijerph18042148 PMC7926308

[5] Kerr J , Anderson C, Lippman SM. Physical activity, sedentary behaviour, diet, and cancer: an update and emerging new evidence. Lancet Oncol. 2017;18:e457–e471. 28759385 10.1016/S1470-2045(17)30411-4 PMC10441558

[6] Mahmood S , MacInnis RJ, English DR, Karahalios A, Lynch BM. Domain-specific physicalactivity and sedentary behaviour in relation to colon and rectal cancer risk: a systematic review and meta-analysis. Int J Epidemiol. 2017;46:1797–1813. 29025130 10.1093/ije/dyx137

[7] Zhang FF , Cudhea F, Shan Z, et al. Preventable cancer burden associated with poor diet in the United States. JNCI Cancer Spectr. 2019;3:pkz034. 31360907 10.1093/jncics/pkz034 PMC6649723

[8] World Health Organisation. Cancer. 2022. https://www.who.int/news-room/fact-sheets/detail/cancer (1 December 2022, date last accessed).

[9] Tech Nation report. 2020. https://technation.io/news/tech-nation-report-2020/. (1 December 2022, date last accessed).

[10] Batty GD , Gale CR, Kivimäki M, Deary IJ, Bell S. Comparison of risk factor associations in UK Biobank against representative, general population based studies with conventional response rates: prospective cohort study and individual participant meta-analysis. BMJ. 2020;368:m131. 32051121 10.1136/bmj.m131 PMC7190071

