# Supplementary material for: Cancer risk in information technology workers: a UK Biobank study
Source: Occup Med (Lond). Author manuscript; Available in PMC 2023 Sep 30. (PMC10540665; doi:10.1093/occmed/kqad070)
Supplement: Supplementary Material [file EMS187525-supplement-Supplementary_Material.doc]

**Supplementary Table 1. Coding of cancer outcomes according to the International Classification of Diseases 9^th^* and 10^th^**** revisions.

| **Cancer site** | **ICD-10 codes** | **ICD-9 codes** |
| --- | --- | --- |
| **All cancers (excluding non- melanoma skin cancer)** | **C00-C97 (excluding C44)** | **140−209 (excluding 173)** |
| **Melanoma** | **C43** | **172** |
| **Oropharyngeal** | **C00-C14** | **140−149** |
| **Lung** | **C33-C34** | **162** |
| **Breast (female only)** | **C50** | **174** |
| **Uterus** | **C54** | **182** |
| **Ovary** | **C56** | **183** |
| **Prostate** | **C61** | **185** |
| **Oesophagus** | **C15** | **150** |
| **Stomach** | **C16** | **151** |
| **Hepatobiliary tract** | **C22-C24** | **155−156** |
| **Pancreatic** | **C25** | **157** |
| **Kidney** | **C64-C65** | **189.0−189.1** |
| **Bladder** | **C66-C67** | **188, 189.2** |
| **Colorectal** | **C18-C21** | **153−154** |
| **Colon** | **C18** | **153** |
| **Rectum** | **C19-C20** | **1540−1541** |
| **Brain tumours** | **C71** | **191** |
| **Thyroid** | **C73** | **193** |
| **Haematological malignancies** | **C81-C96** | **200−208** |
| **Non-Hodgkin's lymphoma** | **C82-C85** | **200, 202** |

*International classification of diseases: [‎9th]‎ ninth revision, basic tabulation list with alphabetic index

<https://apps.who.int/iris/handle/10665/39473>

**International Statistical Classification of Diseases and Related Health Problems (ICD) <https://www.who.int/standards/classifications/classification-of-diseases>

**Supplementary Table 2. Socio-demographic, lifestyle and work characteristics in IT workers compared to the nine major Standard Occupational Classification (SOC) groups**

**in the UK Biobank with cancer registry data linkage.**

|  | All IT workers, n (%) | 1  Other Managers, Directors and Senior Officials | 2  Other Professional Occupations | 3  Other Associate Professional and Technical Occupations | 4 Administrative and Secretarial Occupations | 5  Skilled Trades Occupations | 6  Caring, Leisure and Other Service Occupations | 7  Sales and Customer Service | 8  Process, Plant and Machine Operatives | 9 Elementary Occupations |
| --- | --- | --- | --- | --- | --- | --- | --- | --- | --- | --- |
| Total n (%) 272,733 (100) | 10,517 (4) | 44,080 (16) | 56,565 (21) | 46,748 (17) | 41,200 (15) | 20,285 (7) | 16,889 (6) | 9,442 (3) | 12,447 (5) | 13,765 (5) |
| **Socio-demographic**  Sex  Male  Female  Age (years)  40-44*  45-49  50-54  55-59  60-64  65+  Ethnicity  White  Non-White  Missing**  Townsend deprivation index  1 (least deprived quintile)  2  3  4  5 (most deprived quintile)  Missing**  Household Annual Income (£)  Less than £18 000  £18 000 to £30 999  £31 000 to £51 999  £52 000 to £100 000  Greater than £100 000  Missing**  Highest qualification  Degree,  HNC/HND  School  Other  None of the above  Missing**  **Lifestyle**  Body mass index (kg/m^2^)^1^  < 25  ≥ 25  Missing**  Smoking status  Never smoker  Previous/Current smoker  Missing**  Alcohol consumption^+^(units/week)^2^  ≤ 14  > 14  Missing**  Physical activity (MET min/week)  < 600  ≥ 600  Missing**  Total raw or cooked fruit/vegetables (portions/day)  < 5  ≥ 5  Missing**  Total screen-time outside work^$^ (h/day)  ≤ 2  > 2  Missing**  **Occupational**  Job involves shift work  Never/Rarely  Always/Usually/Sometimes  Missing **  Job involves walking/standing  Always/Usually/Sometimes  Never/Rarely  Missing ** | 8,074 (77)  2,443 (23)  2,593 (25)  2,622 (25)  2,464 (23)  1,782 (17)  917 (9)  139 (1)  9,876 (94)  609 (6)  32 (0)  5,478 (52)  2,331 (22)  1,470 (14)  931 (9)  296 (3)  11 (0)  197 (2)  846 (8)  3,130 (30)  4,552 (43)  1,091 (10)  701 (7)  6,093 (58)  560 (5)  3,518 (34)  96 (1)  132 (1)  118 (1)  3,531 (34)  6,958 (66)  28 (0)  6,589 (63)  3,911 (37)  17 (0)  2,100 (20)  6,027 (57)  2,390 (23)  1,370 (13)  4,512 (43)  4,635 (44)  2,552 (24)  7,867 (75)  98 (1)  5,830 (55)  4,564 (43)  123 (1)  9,773 (93)  738 (7)  6 (0)  2,705 (26)  7,809 (74)  3 (0) | 26,714 (61)  17,366 (39)  7,874 (18)  9,794 (22)  10,031 (23)  8,856 (20)  5,945 (13)  1,580 (4)  41,845 (95)  2,137 (5)  98 (0)  22,312 (51)  9,937 (23)  6,173 (14)  4,147 (9)  1,429 (3)  82 (0)  1,429 (3)  5,112 (12)  12,184 (28)  16,304 (37)  5,669 (13)  3,382 (8)  17,287 (39)  3,101 (7)  18,824 (43)  1,416 (3)  2,917 (7)  535 (1)  12,799 (29)  31,137 (71)  144 (0)  24,320 (55)  19,660 (45)  100 (0)  7,953 (18)  26,539 (60)  9,588 (22)  5,212 (12)  19,556 (44)  19,312 (44)  10,284 (23)  33,233 (75)  563 (1)  22,953 (52)  20,582 (47)  545 (1)  39,112 (89)  4,886 (11)  82 (0)  24,962 (57)  19,072 (43)  46 (0) | 27,802 (49)  28,763 (51)  8,059 (14)  10,116 (18)  12,861 (23)  14,222 (25)  8,905 (16)  2,402 (4)  53,442 (95)  2,959 (5)  164 (0)  28,124 (50)  12,876 (23)  8,250 (15)  5,439 (10)  1,815 (3)  61 (0)  1,571 (3)  6,196 (11)  16,009 (28)  21,888 (39)  7,341 (13)  3,560 (6)  43,667 (77)  1,769 (3)  7,706 (14)  2,431 (4)  415 (1)  577 (1)  22,828 (40)  33,582 (59)  155 (0)  35,807 (63)  20,671 (37)  87 (0)  12,322 (22)  30,945 (55)  13,298 (24)  7,328 (13)  24,804 (44)  24,433 (43)  9,692 (17)  46,319 (82)  554 (1)  35,104 (62)  20,747 (37)  714 (1)  52,700 (93)  3,794 (7)  71 (0)  36,711 (65)  19,825 (35)  29 (0) | 18,904 (40)  27,844 (60)  8,397 (18)  10,512 (22)  10,421 (22)  9,218 (20)  6,385 (14)  1,815 (4)  43,749 (94)  2,836 (6)  163 (0)  21,173 (45)  10,704 (23)  7,230 (15)  5,498 (12)  2,077 (4)  66 (0)  2,765 (6)  9,157 (20)  15,255 (33)  13,113 (28)  2,552 (5)  3,906 (8)  19,094 (41)  2,270 (5)  19,208 (41)  4,142 (9)  1,551 (3)  483 (1)  17,482 (37)  29,117 (62)  149 (0)  26,853 (57)  19,801 (42)  94 (0)  9,633 (21)  23,876 (51)  13,239 (28)  4,710 (10)  21,307 (46)  20,731 (44)  8,575 (18)  37,417 (80)  756 (2)  24,897 (53)  21,157 (45)  694 (2)  35,198 (75)  11,434 (25)  116 (0)  29,852 (64)  16,853 (36)  43 (0) | 8,302 (20)  32,898 (80)  5,793 (14)  8,037 (20)  9,322 (23)  10,000 (24)  6,623 (16)  1,425 (3)  38,997 (95)  2,071 (5)  132 (0)  19,012 (46)  9,591 (23)  6,136 (15)  4,597 (11)  1,812 (4)  52 (0)  4,864 (12)  10,846 (26)  12,331 (30)  7,011 (17)  952 (2)  5,196 (13)  7,437 (18)  1,752 (4)  26,631 (65)  861 (2)  3,927 (9)  592 (1)  15,532 (38)  25,563 (62)  105 (0)  24,932 (61)  16,153 (39)  115 (0)  9,747 (24)  17,066 (41)  14,387 (35)  3,627 (9)  14,322 (35)  23,251 (56)  8,264 (20)  32,097 (78)  839 (2)  18,752 (46)  21,806 (53)  642 (1)  38,146 (93)  3,009 (7)  45 (0)  17,621 (43)  23,547 (57)  32 (0) | 17,991 (89)  2,294 (11)  3,021 (15)  3,929 (19)  4,286 (21)  4,248 (21)  3,841 (19)  960 (5)  19,155 (95)  1,063 (5)  67 (0)  8,390 (41)  4,937 (24)  3,202 (16)  2,694 (13)  1,038 (5)  24 (0)  2,422 (12)  6,031 (30)  6,355 (31)  2,626 (13)  232 (1)  2,619 (13)  2,667 (13)  3,175 (16)  8,939 (44)  1,003 (5)  4,132 (20)  369 (2)  5,194 (26)  15,033 (74)  58 (0)  10,053 (50)  10,164 (50)  68 (0)  3,354 (17)  11,956 (59)  4,975 (25)  964 (5)  11,049 (55)  8,272 (41)  5,229 (26)  14,259 (70)  797 (4)  8,415 (41)  11,463 (57)  407 (2)  15,394 (76)  4,826 (24)  65 (0)  19,001 (94)  1,246 (6)  38 (0) | 2,976 (18)  13,913 (82)  2,732 (16)  3,597 (21)  3,932 (23)  3,634 (22)  2,360 (14)  634 (4)  15,347 (91)  1,476 (9)  66 (0)  6,260 (37)  3,786 (22)  2,805 (17)  2,655 (16)  1,364 (8)  19 (0)  3,849 (23)  4,679 (28)  3,859 (23)  1,755 (10)  206 (1)  2,541 (15)  2,540 (15)  2,726 (16)  8,405 (50)  850 (5)  2,091 (12)  277 (2)  6,050 (36)  10,784 (64)  55 (0)  9,618 (57)  7,200 (43)  71 (0)  3,619 (21)  5,757 (34)  7,513 (45)  1,086 (6)  6,677 (40)  9,126 (54)  3,018 (18)  13,242 (78)  629 (4)  7,509 (45)  8,846 (52)  534 (3)  10,855 (64)  5,950 (35)  84 (0)  15,712 (93)  1,143 (7)  34 (0) | 2,453 (26)  6,989 (74)  1,327 (14)  1,756 (19)  1,970 (21)  2,197 (23)  1,687 (18)  505 (5)  8,708 (92)  711 (8)  23 (0)  3,565 (38)  2,194 (23)  1,601 (17)  1,417 (15)  642 (7)  23 (0)  2,393 (25)  2,621 (28)  1,992 (21)  757 (8)  74 (1)  1,605 (17)  971 (10)  569 (6)  5,023 (53)  161 (2)  2,483 (26)  235 (2)  3,092 (33)  6,328 (67)  22 (0)  5,244 (56)  4,157 (44)  41 (0)  2,013 (21)  3,509 (37)  3,920 (42)  572 (6)  3,298 (35)  5,572 (59)  2,156 (23)  6,938 (73)  348 (4)  3,432 (36)  5,773 (61)  237 (3)  6,979 (74)  2,426 (26)  37 (0)  7,443 (79)  1,986 (21)  13 (0) | 10,984 (88)  1,463 (12)  1,805 (15)  2,368 (19)  2,479 (20)  2,615 (21)  2,594 (21)  586 (5)  11,366 (91)  1,042 (9)  39 (0)  4,095 (33)  2,740 (22)  2,349 (19)  2,203 (18)  1,032 (8)  28 (0)  2,080 (17)  4,076 (33)  3,361 (27)  1,084 (9)  75 (1)  1,771 (14)  1,032 (8)  1,440 (12)  5,345 (43)  469 (4)  3,848 (31)  313 (3)  2,601 (21)  9,798 (79)  48 (0)  5,668 (46)  6,702 (54)  77 (0)  2,074 (17)  6,276 (50)  4,097 (33)  830 (7)  5,405 (43)  6,212 (50)  3,469 (28)  8,252 (66)  726 (6)  4,935 (40)  7,141 (57)  371 (3)  6,884 (55)  5,496 (44)  67 (0)  8,881 (71)  3,508 (28)  58 (0) | 7,567 (55)  6,198 (45)  2,075 (15)  2,613 (19)  2,816 (20)  2,957 (21)  2,624 (19)  680 (5)  12,160 (88)  1,564 (12)  41 (0)  3,680 (27)  2,755 (20)  2,582 (19)  2,978 (22)  1,750 (13)  20 (0)  4,048 (29)  4,167 (30)  2,410 (18)  633 (4.6)  58 (0)  2,449 (18)  1,173 (9)  1,203 (9)  5,753 (42)  264 (2)  4,951 (36)  421 (3)  4,090 (30)  9,612 (70)  63 (0)  6,941 (50)  6,744 (49)  80 (1)  2,456 (18)  5,401 (39)  5,908 (43)  486 (4)  5,725 (42)  7,554 (55)  3,684 (27)  9,152 (66)  929 (7)  4,731 (34)  8,470 (62)  564 (4)  8,910 (65)  4,774 (35)  81 (0)  13,111 (95) 609 (4)  45 (0) |

IQR= interquartile range; HNC= higher national certificate; HND =higher national diploma

*35-39-year-olds added to this total due to very small numbers n=2

MET= metabolic equivalent

** includes ‘missing’, ‘do not know’ and ‘prefer not to answer’ responses

^$^Total screen-time estimated as the sum of computer screen- time outside work and TV viewing (h/day)

^+^Recommended alcohol consumption guidelines changed in 2016 (i.e., following baseline data collection) from 21 units/week for women and 28 units/week for men to current thresholds of 14 units/week for men and women.

**Supplementary Table 3. Cox proportional hazard models of the association between socio-demographic, health, lifestyle and work characteristics and incident cancer∞ in a) IT workers compared to all other employed participants in the UK Biobank and b) IT workers compared to the nine major category standard occupational classification (SOC) occupational groups**

*Longitudinal study population: all employed Biobank parti*cipants with linked national cancer registry data/records

|  |  | **Model 0 ^c^** | **Model 1 ^d^** | **Model 2** ^e^ |
| --- | --- | --- | --- | --- |
|  |  | Unadjusted HR  (95% CI) | Adjusted HR  (95% CI) | Adjusted HR  (95% CI) |
| **a** | Failures 13,351 |  |  |  |
|  | All other employed participants  Incidence rate* (4.7)  All IT workers  Incidence rate* (3.5) | 1.00  0.77  (0.70-0.85) | 1.00  0.91  (0.83-1.01) | 1.00  0.92  (0.83-1.02) |
| **b1** | Failures 1,082 |  |  |  |
|  | All other Managers, Directors and Senior Officials  Incidence rate* (4.8)  All IT workers  Incidence rate* (3.5) | 1.00  0.77  (0.69-0.86) | 1.00  0.89  (0.80-1.00) | 1.00  0.91  (0.80-1.03) |
| **b2** | Failures 1,405 |  |  |  |
|  | All other Professional Occupations Incidence rate* (4.7)  All IT workers  Incidence rate* (3.5) | 1.00  0.76  (0.68-0.84) | 1.00  0.97  (0.87-1.08) | 1.00  0.94  (0.83-1.07) |
| **b3** | Failures 1,045 |  |  |  |
|  | All other Associate Professional and Technical Occupations  Incidence rate* (4.4)  All IT workers  Incidence rate* (3.5) | 1.00  0.81  (0.73-0.90) | 1.00  0.90  (0.80-1.00) | 1.00  0.87  (0.76-1.00) |
| **b4** | Failures 715 |  |  |  |
|  | Administrative and Secretarial Occupations  Incidence rate* (4.7)  All IT workers  Incidence rate* (3.5) | 1.00  0.77  (0.69-0.86) | 1.00  0.90  (0.80-1.03) | 1.00  1.03  (0.87-1.21) |
| **b5** | Failures 648 |  |  |  |
|  | Skilled Trades Occupations Incidence rate* (4.6)  All IT workers  Incidence rate* (3.5) | 1.00  0.75  (0.67-0.84) | 1.00  1.02  (0.89-1.16) | 1.00  0.97  (0.80-1.19) |
| **b6** | Failures 356 |  |  |  |
|  | Caring, Leisure and Other Service Occupations  Incidence rate* (4.3)  All IT workers  Incidence rate* (3.5) | 1.00  0.84  (0.74-0.95) | 1.00  0.94  (0.80-1.10) | 1.00  1.09  (0.84-1.40) |
| **b7** | Failures 293 |  |  |  |
|  | Sales and Customer Service Occupations  Incidence rate* (4.6)  All IT workers  Incidence rate* (3.5) | 1.00  0.72  (0.57-0.91) | 1.00  0.86  (0.64-1.16) | 1.00  0.79  (0.57-1.09) |
| **b8** | Failures 371 |  |  |  |
|  | Process, Plant and Machine Operatives  Incidence rate* (5.2)  All IT workers  Incidence rate* (3.5) | 1.00  0.71  (0.63-0.80) | 1.00  0.98  (0.84-1.14) | 1.00  1.14  (0.91-1.44) |
| **b9** | Failures 385 |  |  |  |
|  | Elementary Occupations  Incidence rate* (4.9)  All IT workers  Incidence rate* (3.5) | 1.00  0.70  (0.62-0.79) | 1.00  0.92  (0.78-1.07) | 1.00  0.87  (0.68-1.12) |

HR, hazard ratio; CI, confidence interval.

Model 0 ^c^ = Unadjusted

Model 1 ^d^ = Model 0 + potential confounders/socio-demographic factors i.e., age, sex, ethnicity, deprivation index, educational attainment, assessment centre, date of assessment

Model 2 ^e^ = Model 1 + potential mediators/ lifestyle factors i.e., smoking status, alcohol consumption, fruit and vegetable consumption, BMI, physical activity, total screen-time outside work (tv viewing time, non-work computer use), shift work, work-related sedentary behaviour

*Rates are expressed per 1000 and based on person-years

∞Cancer registry data were available from 1957 onwards until 31 January 2021 for Scotland and from 1971 onwards until 29 February 2020 for England & Wales.

**Figure 1. Flow chart of the selection process** **and samples included in the longitudinal analyses**

**Participants who completed the UK Biobank (UKB) computer assisted self-administered questionnaire at baseline n=502,412***

**Currently employed**

**All employed participants in UKB at baseline for whom cancer registry data were also available n=287,085**

**Unemployed participants in UKB at baseline**

**n=215,327**

**14,352 participants with pre-existing cancer at and pre UKB baseline years excluded.**

**All employed participants without pre-existing cancer at and pre UKB baseline n=** **272,733**

**IT workers n=** **10,517**

**All other employed participants n=** **262,216**

**SOC MAJOR GROUPS (missing=795)**

**n=287,141**

**Administrative and Secretarial Occupations**

**n=41,200**

**Other Associate Professional and Technical Occupations**

**n=46,748**

**Caring, Leisure and Other Service Occupations**

**n=16,889**

**Other Managers, Directors and Senior Officials**

**n=44,080**

**Sales and Customer Service Occupations**

**n=9,442**

**Process, Plant and Machine Operatives**

**n=12,447**

**Elementary Occupations**

**n=13,765**

**Skilled Trades Occupations**

**n=20,285**

**Other Professional Occupations**

**n=56,565**

*UK Biobank data accessed on 25.02.22
