# Supplementary material for: Cancer risk in information technology workers: a UK Biobank study
Source: Occup Med (Lond). 2023 Jul 21;73(6):304–8. doi: 10.1093/occmed/kqad070 (PMC10540665; doi:10.1093/occmed/kqad070)
Supplement: Supplementary file 1 [file kqad070_suppl_supplementary_material.doc]

Supplementary Table 1. Coding of cancer outcomes according to the International Classification of Diseases 9th* and 10th revisions.**

Cancer site	ICD-10 codes	ICD-9 codes
All cancers (excluding non-melanoma skin cancer)	C00-C97 (excluding C44)	140−209 (excluding 173)
Melanoma	C43	172
Oropharyngeal	C00-C14	140−149
Lung	C33-C34	162
Breast (female only)	C50	174
Uterus	C54	182
Ovary	C56	183
Prostate	C61	185
Oesophagus	C15	150
Stomach	C16	151
Hepatobiliary tract	C22-C24	155−156
Pancreatic	C25	157
Kidney	C64-C65	189.0−189.1
Bladder	C66-C67	188, 189.2
Colorectal	C18-C21	153−154
Colon	C18	153
Rectum	C19-C20	1540−1541
Brain tumours	C71	191
Thyroid	C73	193
Haematological malignancies	C81-C96	200−208
Non-Hodgkin's lymphoma	C82-C85	200, 202

*International classification of diseases: [9th] ninth revision, basic tabulation list with alphabetic index
https://apps.who.int/iris/handle/10665/39473
**International Statistical Classification of Diseases and Related Health Problems (ICD)
https://www.who.int/standards/classifications/classification-of-diseases

Supplementary Table 2. Socio-demographic, lifestyle and work characteristics in IT workers compared to the nine major Standard Occupational Classification (SOC) groups in the UK Biobank with cancer registry data linkage.

	All IT workers, n (%)	1 Other Managers, Directors and Senior Officials	2 Other Professional Occupations	3 Other Associate Professional and Technical Occupations	4 Administrative and Secretarial Occupations	5 Skilled Trades Occupations	6 Caring, Leisure and Other Service Occupations	7 Sales and Customer Service	8 Process, Plant and Machine Operatives	9 Elementary Occupations
Total n (%) 272,733 (100)	10,517 (4)	44,080 (16)	56,565 (21)	46,748 (17)	41,200 (15)	20,285 (7)	16,889 (6)	9,442 (3)	12,447 (5)	13,765 (5)
**Socio-demographic**										
Sex										
Male	8,074 (77)	26,714 (61)	27,802 (49)	18,904 (40)	8,302 (20)	17,991 (89)	2,976 (18)	2,453 (26)	10,984 (88)	7,567 (55)
Female	2,443 (23)	17,366 (39)	28,763 (51)	27,844 (60)	32,898 (80)	2,294 (11)	13,913 (82)	6,989 (74)	1,463 (12)	6,198 (45)
Age (years)										
40-44*	2,593 (25)	7,874 (18)	8,059 (14)	8,397 (18)	5,793 (14)	3,021 (15)	2,732 (16)	1,327 (14)	1,805 (15)	2,075 (15)
45-49	2,622 (25)	9,794 (22)	10,116 (18)	10,512 (22)	8,037 (20)	3,929 (19)	3,597 (21)	1,756 (19)	2,368 (19)	2,613 (19)
50-54	2,464 (23)	10,031 (23)	12,861 (23)	10,421 (22)	9,322 (23)	4,286 (21)	3,932 (23)	1,970 (21)	2,479 (20)	2,816 (20)
55-59	1,782 (17)	8,856 (20)	14,222 (25)	9,218 (20)	10,000 (24)	4,248 (21)	3,634 (22)	2,197 (23)	2,615 (21)	2,957 (21)
60-64	917 (9)	5,945 (13)	8,905 (16)	6,385 (14)	6,623 (16)	3,841 (19)	2,360 (14)	1,687 (18)	2,594 (21)	2,624 (19)
65+	139 (1)	1,580 (4)	2,402 (4)	1,815 (4)	1,425 (3)	960 (5)	634 (4)	505 (5)	586 (5)	680 (5)
Ethnicity										
White	9,876 (94)	41,845 (95)	53,442 (95)	43,749 (94)	38,997 (95)	19,155 (95)	15,347 (91)	8,708 (92)	11,366 (91)	12,160 (88)
Non-White	609 (6)	2,137 (5)	2,959 (5)	2,836 (6)	2,071 (5)	1,063 (5)	1,476 (9)	711 (8)	1,042 (9)	1,564 (12)
Missing**	32 (0)	98 (0)	164 (0)	163 (0)	132 (0)	67 (0)	66 (0)	23 (0)	39 (0)	41 (0)
Townsend deprivation index										
1 (least deprived quintile)	5,478 (52)	22,312 (51)	28,124 (50)	21,173 (45)	19,012 (46)	8,390 (41)	6,260 (37)	3,565 (38)	4,095 (33)	3,680 (27)
2	2,331 (22)	9,937 (23)	12,876 (23)	10,704 (23)	9,591 (23)	4,937 (24)	3,786 (22)	2,194 (23)	2,740 (22)	2,755 (20)
3	1,470 (14)	6,173 (14)	8,250 (15)	7,230 (15)	6,136 (15)	3,202 (16)	2,805 (17)	1,601 (17)	2,349 (19)	2,582 (19)
4	931 (9)	4,147 (9)	5,439 (10)	5,498 (12)	4,597 (11)	2,694 (13)	2,655 (16)	1,417 (15)	2,203 (18)	2,978 (22)
5 (most deprived quintile)	296 (3)	1,429 (3)	1,815 (3)	2,077 (4)	1,812 (4)	1,038 (5)	1,364 (8)	642 (7)	1,032 (8)	1,750 (13)
Missing**	11 (0)	82 (0)	61 (0)	66 (0)	52 (0)	24 (0)	19 (0)	23 (0)	28 (0)	20 (0)
Household Annual Income (£)										
Less than £18 000	197 (2)	1,429 (3)	1,571 (3)	2,765 (6)	4,864 (12)	2,422 (12)	3,849 (23)	2,393 (25)	2,080 (17)	4,048 (29)
£18 000 to £30 999	846 (8)	5,112 (12)	6,196 (11)	9,157 (20)	10,846 (26)	6,031 (30)	4,679 (28)	2,621 (28)	4,076 (33)	4,167 (30)
£31 000 to £51 999	3,130 (30)	12,184 (28)	16,009 (28)	15,255 (33)	12,331 (30)	6,355 (31)	3,859 (23)	1,992 (21)	3,361 (27)	2,410 (18)
£52 000 to £100 000	4,552 (43)	16,304 (37)	21,888 (39)	13,113 (28)	7,011 (17)	2,626 (13)	1,755 (10)	757 (8)	1,084 (9)	633 (4.6)
Greater than £100 000	1,091 (10)	5,669 (13)	7,341 (13)	2,552 (5)	952 (2)	232 (1)	206 (1)	74 (1)	75 (1)	58 (0)
Missing**	701 (7)	3,382 (8)	3,560 (6)	3,906 (8)	5,196 (13)	2,619 (13)	2,541 (15)	1,605 (17)	1,771 (14)	2,449 (18)
Highest qualification										
Degree,	6,093 (58)	17,287 (39)	43,667 (77)	19,094 (41)	7,437 (18)	2,667 (13)	2,540 (15)	971 (10)	1,032 (8)	1,173 (9)
HNC/HND	560 (5)	3,101 (7)	1,769 (3)	2,270 (5)	1,752 (4)	3,175 (16)	2,726 (16)	569 (6)	1,440 (12)	1,203 (9)
School	3,518 (34)	18,824 (43)	7,706 (14)	19,208 (41)	26,631 (65)	8,939 (44)	8,405 (50)	5,023 (53)	5,345 (43)	5,753 (42)
Other	96 (1)	1,416 (3)	2,431 (4)	4,142 (9)	861 (2)	1,003 (5)	850 (5)	161 (2)	469 (4)	264 (2)
None of the above	132 (1)	2,917 (7)	415 (1)	1,551 (3)	3,927 (9)	4,132 (20)	2,091 (12)	2,483 (26)	3,848 (31)	4,951 (36)
Missing**	118 (1)	535 (1)	577 (1)	483 (1)	592 (1)	369 (2)	277 (2)	235 (2)	313 (3)	421 (3)
**Lifestyle**										
Body mass index (kg/m^2)[1]										
< 25	3,531 (34)	12,799 (29)	22,828 (40)	17,482 (37)	15,532 (38)	5,194 (26)	6,050 (36)	3,092 (33)	2,601 (21)	4,090 (30)
≥ 25	6,958 (66)	31,137 (71)	33,582 (59)	29,117 (62)	25,563 (62)	15,033 (74)	10,784 (64)	6,328 (67)	9,798 (79)	9,612 (70)
Missing**	28 (0)	144 (0)	155 (0)	149 (0)	105 (0)	58 (0)	55 (0)	22 (0)	48 (0)	63 (0)
Smoking status										

Never smoker	6,589 (63)	24,320 (55)	35,807 (63)	26,853 (57)	24,932 (61)	10,053 (50)	9,618 (57)	5,244 (56)	5,668 (46)	6,941 (50)
Previous/Current smoker	3,911 (37)	19,660 (45)	20,671 (37)	19,801 (42)	16,153 (39)	10,164 (50)	7,200 (43)	4,157 (44)	6,702 (54)	6,744 (49)
Missing**	17 (0)	100 (0)	87 (0)	94 (0)	115 (0)	68 (0)	71 (0)	41 (0)	77 (0)	80 (1)
Alcohol consumption[+](units/week)[2]										
≤ 14	2,100 (20)	7,953 (18)	12,322 (22)	9,633 (21)	9,747 (24)	3,354 (17)	3,619 (21)	2,013 (21)	2,074 (17)	2,456 (18)
> 14	6,027 (57)	26,539 (60)	30,945 (55)	23,876 (51)	17,066 (41)	11,956 (59)	5,757 (34)	3,509 (37)	6,276 (50)	5,401 (39)
Missing**	2,390 (23)	9,588 (22)	13,298 (24)	13,239 (28)	14,387 (35)	4,975 (25)	7,513 (45)	3,920 (42)	4,097 (33)	5,908 (43)
Physical activity (MET min/week)										
< 600	1,370 (13)	5,212 (12)	7,328 (13)	4,710 (10)	3,627 (9)	964 (5)	1,086 (6)	572 (6)	830 (7)	486 (4)
≥ 600	4,512 (43)	19,556 (44)	24,804 (44)	21,307 (46)	14,322 (35)	11,049 (55)	6,677 (40)	3,298 (35)	5,405 (43)	5,725 (42)
Missing**	4,635 (44)	19,312 (44)	24,433 (43)	20,731 (44)	23,251 (56)	8,272 (41)	9,126 (54)	5,572 (59)	6,212 (50)	7,554 (55)
Total raw or cooked fruit/vegetables (portions/day)										
< 5	2,552 (24)	10,284 (23)	9,692 (17)	8,575 (18)	8,264 (20)	5,229 (26)	3,018 (18)	2,156 (23)	3,469 (28)	3,684 (27)
≥ 5	7,867 (75)	33,233 (75)	46,319 (82)	37,417 (80)	32,097 (78)	14,259 (70)	13,242 (78)	6,938 (73)	8,252 (66)	9,152 (66)
Missing**	98 (1)	563 (1)	554 (1)	756 (2)	839 (2)	797 (4)	629 (4)	348 (4)	726 (6)	929 (7)
Total screen-time outside work[$] (h/day)										
≤ 2	5,830 (55)	22,953 (52)	35,104 (62)	24,897 (53)	18,752 (46)	8,415 (41)	7,509 (45)	3,432 (36)	4,935 (40)	4,731 (34)
> 2	4,564 (43)	20,582 (47)	20,747 (37)	21,157 (45)	21,806 (53)	11,463 (57)	8,846 (52)	5,773 (61)	7,141 (57)	8,470 (62)
Missing**	123 (1)	545 (1)	714 (1)	694 (2)	642 (1)	407 (2)	534 (3)	237 (3)	371 (3)	564 (4)
**Occupational**										
Job involves shift work										
Never/Rarely	9,773 (93)	39,112 (89)	52,700 (93)	35,198 (75)	38,146 (93)	15,394 (76)	10,855 (64)	6,979 (74)	6,884 (55)	8,910 (65)
Always/Usually/Sometimes	738 (7)	4,886 (11)	3,794 (7)	11,434 (25)	3,009 (7)	4,826 (24)	5,950 (35)	2,426 (26)	5,496 (44)	4,774 (35)
Missing **	6 (0)	82 (0)	71 (0)	116 (0)	45 (0)	65 (0)	84 (0)	37 (0)	67 (0)	81 (0)
Job involves walking/standing										
Always/Usually/Sometimes	2,705 (26)	24,962 (57)	36,711 (65)	29,852 (64)	17,621 (43)	19,001 (94)	15,712 (93)	7,443 (79)	8,881 (71)	13,111 (95)
Never/Rarely	7,809 (74)	19,072 (43)	19,825 (35)	16,853 (36)	23,547 (57)	1,246 (6)	1,143 (7)	1,986 (21)	3,508 (28)	609 (4)
Missing **	3 (0)	46 (0)	29 (0)	43 (0)	32 (0)	38 (0)	34 (0)	13 (0)	58 (0)	45 (0)

IQR= interquartile range; HNC= higher national certificate; HND =higher national diploma
*35-39-year-olds added to this total due to very small numbers n=2
MET= metabolic equivalent
** includes 'missing', 'do not know' and 'prefer not to answer' responses
[$]Total screen-time estimated as the sum of computer screen- time outside work and TV viewing (h/day)
[+]Recommended alcohol consumption guidelines changed in 2016 (i.e., following baseline data collection) from 21 units/week for women and 28 units/week for men to current thresholds of 14 units/week for men and women.

Supplementary Table 3. Cox proportional hazard models of the association between socio-demographic, health, lifestyle and work characteristics and incident cancer∞ in a) IT workers compared to all other employed participants in the UK Biobank and b) IT workers compared to the nine major category standard occupational classification (SOC) occupational groups

*Longitudinal study population: all employed Biobank parti*cipants with linked national cancer registry data/records

		**Model 0** [c]	**Model 1** [d]	**Model 2** [e]
		Unadjusted HR (95% CI)	Adjusted HR (95% CI)	Adjusted HR (95% CI)
**a**	Failures 13,351			
	All other employed participants<br>Incidence rate* (4.7)	1.00	1.00	1.00
	All IT workers<br>Incidence rate* (3.5)	0.77<br>(0.70-0.85)	0.91<br>(0.83-1.01)	0.92<br>(0.83-1.02)
**b1**	Failures 1,082			
	All other Managers, Directors and Senior Officials<br>Incidence rate* (4.8)	1.00	1.00	1.00
	All IT workers<br>Incidence rate* (3.5)	0.77<br>(0.69-0.86)	0.89<br>(0.80-1.00)	0.91<br>(0.80-1.03)
**b2**	Failures 1,405			
	All other Professional Occupations<br>Incidence rate* (4.7)	1.00	1.00	1.00
	All IT workers<br>Incidence rate* (3.5)	0.76<br>(0.68-0.84)	0.97<br>(0.87-1.08)	0.94<br>(0.83-1.07)
**b3**	Failures 1,045			
	All other Associate Professional and Technical Occupations<br>Incidence rate* (4.4)	1.00	1.00	1.00
	All IT workers<br>Incidence rate* (3.5)	0.81<br>(0.73-0.90)	0.90<br>(0.80-1.00)	0.87<br>(0.76-1.00)
**b4**	Failures 715			
	Administrative and Secretarial Occupations<br>Incidence rate* (4.7)	1.00	1.00	1.00
	All IT workers<br>Incidence rate* (3.5)	0.77<br>(0.69-0.86)	0.90<br>(0.80-1.03)	1.03<br>(0.87-1.21)

**b5**	Failures 648			
	Skilled Trades Occupations<br>Incidence rate* (4.6)<br><br>All IT workers<br>Incidence rate* (3.5)	1.00<br><br><br>0.75<br>(0.67-0.84)	1.00<br><br><br>1.02<br>(0.89-1.16)	1.00<br><br><br>0.97<br>(0.80-1.19)
**b6**	Failures 356			
	Caring, Leisure and Other Service Occupations<br>Incidence rate* (4.3)<br><br>All IT workers<br>Incidence rate* (3.5)	1.00<br><br><br>0.84<br>(0.74-0.95)	1.00<br><br><br>0.94<br>(0.80-1.10)	1.00<br><br><br>1.09<br>(0.84-1.40)
**b7**	Failures 293			
	Sales and Customer Service Occupations<br>Incidence rate* (4.6)<br><br>All IT workers<br>Incidence rate* (3.5)	1.00<br><br><br>0.72<br>(0.57-0.91)	1.00<br><br><br>0.86<br>(0.64-1.16)	1.00<br><br><br>0.79<br>(0.57-1.09)
**b8**	Failures 371			
	Process, Plant and Machine Operatives<br>Incidence rate* (5.2)<br><br>All IT workers<br>Incidence rate* (3.5)	1.00<br><br><br>0.71<br>(0.63-0.80)	1.00<br><br><br>0.98<br>(0.84-1.14)	1.00<br><br><br>1.14<br>(0.91-1.44)
**b9**	Failures 385			
	Elementary Occupations<br>Incidence rate* (4.9)<br><br>All IT workers<br>Incidence rate* (3.5)	1.00<br><br><br>0.70<br>(0.62-0.79)	1.00<br><br><br>0.92<br>(0.78-1.07)	1.00<br><br><br>0.87<br>(0.68-1.12)

HR, hazard ratio; CI, confidence interval.
Model 0 [c] = Unadjusted
Model 1 [d] = Model 0 + potential confounders/socio-demographic factors i.e., age, sex, ethnicity, deprivation index, educational attainment, assessment centre, date of assessment
Model 2 [e] = Model 1 + potential mediators/ lifestyle factors i.e., smoking status, alcohol consumption, fruit and vegetable consumption, BMI, physical activity, total screen-time outside work (tv viewing time, non-work computer use), shift work, work-related sedentary behaviour
*Rates are expressed per 1000 and based on person-years
∞Cancer registry data were available from 1957 onwards until 31 January 2021 for Scotland and from 1971 onwards until 29 February 2020 for England & Wales.

Figure 1. Flow chart of the selection process and samples included in the longitudinal analyses

Participants who completed the UK Biobank (UKB) computer assisted self-administered questionnaire at baseline n=502,412*

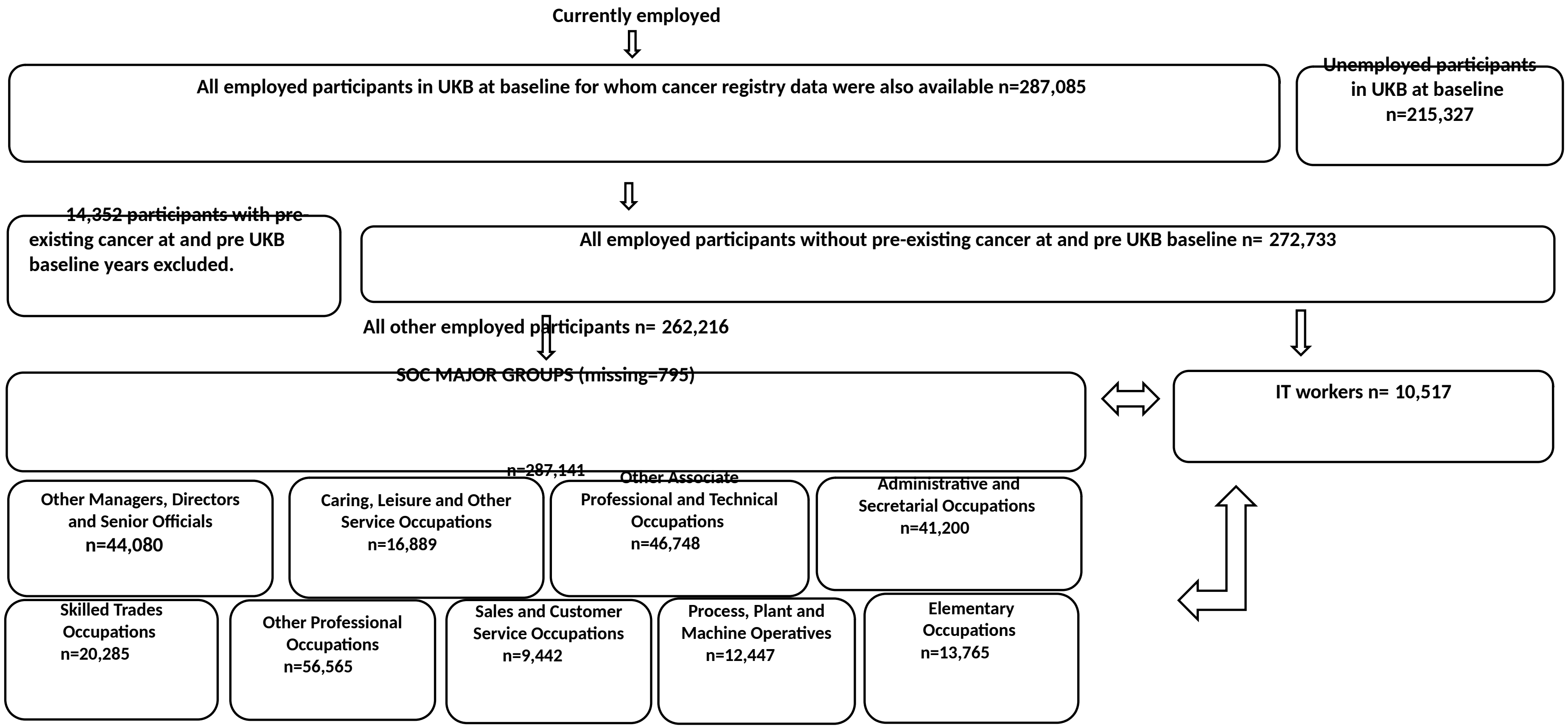

*UK Biobank data accessed on 25.02.22
